# Developing and implementing a point-of-care cardiac ultrasound teaching module for medical students: perceptions, needs, and outcomes

**DOI:** 10.3389/fmed.2026.1797086

**Published:** 2026-04-29

**Authors:** Sulafa K. M. Ali, Rania S. Ahmed, Mohamed Hassan Taha

**Affiliations:** 1 Department of Clinical Sciences, College of Medicine, University of Sharjah, Sharjah, United Arab Emirates; 2 Department of Basic Sciences, College of Medicine, University of Sharjah, Sharjah, United Arab Emirates; 3 Department of Medical Education, College of Medicine, University of Sharjah, Sharjah, United Arab Emirates

**Keywords:** Cardiac, point-of-care, ultrasound, medical, education

## Abstract

**Introduction:**

Point-of-care ultrasound (POCUS) has become an essential diagnostic tool in clinical practice, particularly in the emergency room. While POCUS has recently been incorporated into medical school curricula, the introduction of specialized cardiac POCUS (cPOCUS) curricula remains limited. This study aimed to develop, implement, and evaluate a cPOCUS module for medical students.

**Methods:**

A single group pre/post educational intervention study was conducted at the University of Sharjah, United Arab Emirates, in November 2025. Participants included year 4 medical students. Students’ perceptions, prior exposure, and learning needs regarding point-of-care ultrasound were assessed. A structured 5-hour cPOCUS teaching module was implemented, including theoretical and hands-on sessions. Outcomes were assessed using multiple-choice questions (MCQs) and a practical skills checklist.

**Results and discussion:**

While all students expressed a motivation to learn POCUS and 90% considered it essential for emergency care, only 17% had prior exposure to POCUS training. Following the module, the median knowledge score increased from 4 (IQR: 2-8) to 8 (IQR: 6.75-9) (*p* < 0.05). The practical skills assessment showed “very good” performance in 94% of participants. Almost all students (95%) felt confident performing cPOCUS. This module enhanced theoretical understanding and practical skills, fostering a high level of confidence in imaging in the short term. These findings support the integration of cPOCUS in undergraduate medical school curricula.

## 1 Introduction

Ultrasound (US) technology is rapidly advancing with the recent launch of small portable machines. Handheld US device innovation marked the era of point-of-care US (POCUS), defined as a focused, bedside US study performed by frontline healthcare staff with targeted training (1). Its utility expanded to include almost all medical conditions, both for diagnostic and interventional purposes, such as guiding intravenous line insertion and pericardiocentesis (2, 3). Cardiac POCUS (cPOCUS) shortens time to diagnosis and improves diagnostic accuracy for patients with heart disease presenting to the emergency department. Emergency conditions such as heart failure and pericardial effusion can be readily diagnosed at the bedside using limited views, thus enabling timely intervention management (4). POCUS training has been introduced into medical curricula since 2005. Teaching cPOCUS to students during the clinical phase has been shown to significantly improve the accuracy of bedside diagnosis (5, 6). Additionally, POCUS has been incorporated into pre-clinical students’ curricula to augment anatomy sessions and has proven to be an effective method for teaching living clinical anatomy (7, 8).

Despite its proven clinical utility (2–4), POCUS education is still underrepresented in undergraduate medical curricula. Only one-third of countries in the European Union have integrated POCUS into the undergraduate curriculum; furthermore, most of these courses are introductory and do not aim to transition students toward autonomous clinical practice (9). These findings expose significant gaps in medical education and highlight the need for studies that assess the implementation and efficacy of POCUS training modules. This study aimed to assess undergraduate medical students’ baseline perceptions, prior exposure, and learning needs regarding point-of-care ultrasound, and to evaluate the effectiveness and feasibility of implementing a structured cardiac POCUS (cPOCUS) teaching module in improving their theoretical knowledge and basic practical skills.

## 2 Methodology

This is a single group pre/post educational intervention study conducted at the University of Sharjah, United Arab Emirates, from 15–30 November 2025, for Year 4 students undertaking a clinical clerkship.

The study team developed research instruments, including a questionnaire, videos, and a checklist. Content validity was established through an expert panel review and pretesting of the questionnaire, which assessed the relevance and utility of each item and systematically evaluated each item’s relevance, clarity, and practical utility.

Assessment domains and corresponding methods are summarized in Table 1.

**Table 1:** Module domains, learning objectives, and assessment methods.

Domain	Learning objectives	Assessment method
Professional values, attitudes, and ethics	To practice professionalism in dealing with patients/subjects, commitment to time management	Practical assessment (observation)
Theoretical scientific content	To understand the basic principles of ultrasound physics and the operation of the cPOCUS machine To know the clinical indications for cPOCUS and understand its limitations To recognize the basic cPOCUS views and their interpretation	MCQs and recorded image interpretation
Practical skills	To acquire the basic cPOCUS views, including the subcostal, parasternal long-axis, parasternal short-axis, and apical four-chamber views To know how to interpret normal and common abnormal findings on cPOCUS examination	Practical assessment using a checklist and recorded images

The Kern’s six-step approach to curriculum development was used to design the program and to guide the implementation of the study. Kern’s model provides a systematic framework for designing, implementing, and evaluating medical education programs (10). A structured questionnaire was created to identify general needs assessment and curricular gaps, as well as general and targeted needs related to cPOCUS. All Year 4 medical students were invited to participate in the study. The questionnaire was distributed electronically to all Year 4 medical students before the module was implemented. The questionnaire explored students’ prior knowledge of ultrasound, exposure to POCUS during clinical training, practical experience with ultrasound machines, and their perceptions regarding including cPOCUS in the undergraduate curriculum. Based on the needs assessments, the module’s educational goal was to equip students with basic competency in cPOCUS. These objectives and assessment methods are presented in Table 1.

A blended instructional strategy was employed to support progressive learning and skills development. Students were first provided with structured handouts for 1 hour of self-directed study covering core information on ultrasound principles, machine features, indications, and limitations of cPOCUS. The handouts also included annotated images illustrating the basic cardiac views used in cPOCUS. In addition, students had access to a recorded demonstration video created by the study author that illustrated the practical steps involved in performing cPOCUS, including probe positioning and image acquisition for each standard view. To assess baseline knowledge and stimulate engagement, students completed a pre-lecture multiple-choice questionnaire (MCQ) comprising 10 items. Knowledge levels were categorized as poor (score <5), good (score 5–6), and very good (score >6). A 45-min online lecture was delivered, focusing on ultrasound physics, scanning techniques, and the indications and limitations of cPOCUS. The lecture incorporated live ultrasound images demonstrating normal cardiac views, together with recorded examples of common abnormal findings. Following the lecture, students completed a post-lecture MCQ assessment, similar in structure and content to the pre-assessment, along with an interpretation of five recorded echocardiography videos representing both normal and abnormal studies, tested using MCQ format. To support effective skills training, three medical tutors were recruited to assist with student instruction. These tutors were provided with the same theoretical teaching materials and demonstration video as the students. In addition, they completed an 8-h hands-on practical training session using volunteer subjects to ensure consistency in instruction and supervision. The practical component utilized two Clarius^®^ handheld ultrasound machines. These wireless devices display images on tablet and mobile phone screens and support two-dimensional imaging, color flow mapping, pulse Doppler, and M-mode. A standardized cardiac ultrasound protocol of the American Society of Echocardiography was followed (11). Four core cPOCUS views were demonstrated and practiced: the subcostal view; parasternal long-axis view with and without color flow mapping; parasternal short-axis view with and without M-mode; and the apical four-chamber view with and without color flow mapping. After completing the theoretical component, students attended a 4-h hands-on practical workshop conducted using human volunteers. Training was delivered to small groups to maximize hands-on exposure. Trainers demonstrated each view before students practiced image acquisition under direct supervision, with real-time feedback on probe handling, image optimization, and interpretation. At the conclusion of the workshop, the first author and trained tutors assessed students’ practical skills using a structured checklist that evaluated image acquisition, image quality, and interpretation (12). There were 5 items: 4 for image acquisition (all views obtained, views clearly shown, all intended structures shown) and 1 for view interpretation. Each item was scored on a three-point scale: 0 (not performed), 1 (partial performance), and 2 (good performance), yielding a total score out of 10. Overall performance was graded as excellent (8–10), good (5–8), or poor (<5).

Upon completion of the module, students completed a post-implementation questionnaire on a 5-point Likert scale to evaluate their learning experience and satisfaction with the module. This questionnaire contained open-ended questions. Likert data were analyzed as ordinal variables. Free-text responses were analyzed using thematic coding followed by categorization and frequency reporting.

### 2.1 Statistical analysis

Collected data were tabulated and analyzed using the Statistical Package for Social Sciences (SPSS), version 26 (SPSS Inc., Chicago, IL, United States). Qualitative variables were expressed as frequencies and percentages, while quantitative variables were summarized using mean, standard deviation, median, and range. The Wilcoxon signed-rank test was used to compare the median knowledge score data before and after the lecture. A *p*-value <0.05 was considered statistically significant.

## 3 Results

**Figure 1:**
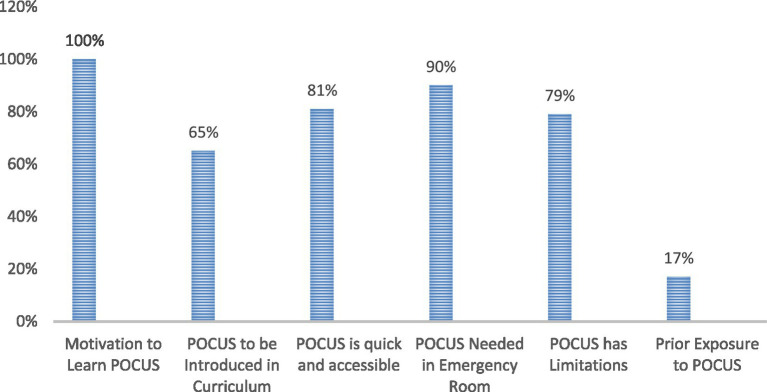
Students’ perceptions, needs, and prior training regarding POCUS (%).

**Figure 2:**
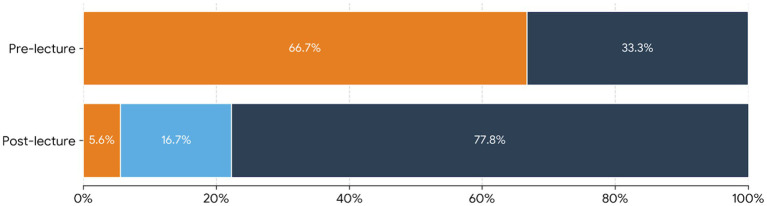
Students’ knowledge scores (%) pre and post lecture (20 students). 
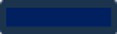
 Very good. 
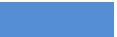
 Good. 
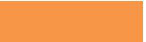
 Poor.

A total of 47 out of 122 Year 4 medical students responded to the Need Assessment survey, and the first 20 students who provided written consent were enrolled in module implementation. The students had 6 weeks of clinical training before the POCUS course. Perceptions and prior exposure to POCUS training are shown in Figure 1. Figure 2 shows the knowledge scores before and after the theoretical module. All 20 students completed all the parts of the module. Knowledge scores improved significantly following the theoretical module. The median knowledge score increased from 4 (IQR: 2–8) at baseline to 8 (IQR: 6.75–9) post intervention (Wilcoxon signed-rank test *p* < 0.05).

The recorded videos test showed a median score of 81 (IQR: 74–88).

The overall practical performance score (out of 10) was good (5–6) in 4.8% and very good (>6) in 94.2% of students. The median performance score (IQR) = 9 (8–10).

**Figure 3:**
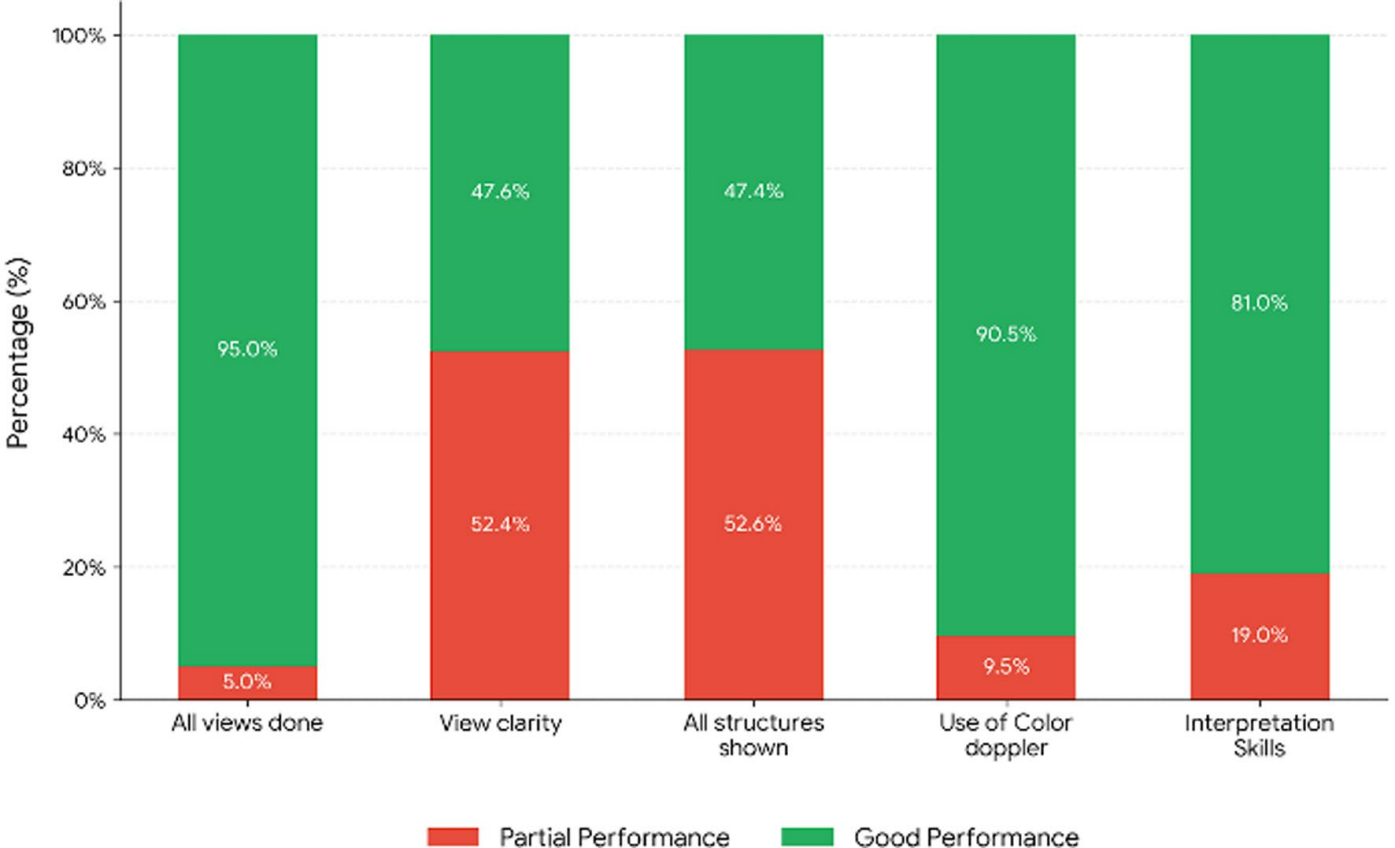
Students’ practical skills assessment of 5 items: all views obtained, views clearly shown, all intended structures shown, use of Doppler, and view interpretation (number of students = 20).

Figure 3 shows the detailed performance in each of the 5 practical items.

All students showed professional attitudes while dealing with the volunteer subjects. The students’ satisfaction questionnaire was completed by all 20 students at the end of the module shown in Table 2. Students reported that the lecture and practical sessions were well explained, and the hands-on practical teaching was most valuable. They suggested including a wider range of normal and abnormal patients to provide greater training exposure.

**Table 2:** Student satisfaction assessment (all 20 students answered each question).

Question	Response %				Strongly disagree
	Strongly agree	Agree	Neutral	Disagree	
The overall experience with the cPOCUS workshop was useful	85	10	0	5	0
The module met your expectations	100	0	0		0
You feel confident in performing basic cPOCUS views after the workshop	65	30	5	0	0
The theoretical content (handout and lecture) was clear and informative	95	5	0	0	0
The practical session was well-organized and effective	75	20	5	0	0

## 4 Discussion

As POCUS becomes a well-established clinical tool, most students who responded to the Need Assessment Questionnaire in this study acknowledged its vital role in clinical practice, particularly in the emergency room. The study uncovered significant knowledge and training gaps. The most obvious gap is that most students lacked prior exposure to POCUS training. This deficit underscores the critical need to integrate POCUS training in the formal medical curricula. This necessity was formalized during a consensus meeting involving 64 multidisciplinary US experts, resulting in a joint recommendation to integrate POCUS into the undergraduate medical curriculum (13).

A recent survey of US medical schools identifies emergency and cardiac POCUS programs as the most taught modules, reflecting their high clinical demand and diagnostic impact (14).

All our students expressed a strong motivation to receive POCUS training, acknowledging the growing role of ultrasound in modern medical practice. This structured cPOCUS teaching module demonstrated that a short, well-designed educational intervention can lead to significant improvements in students’ theoretical knowledge, basic scanning skills, and image interpretation. Students’ knowledge of techniques, indications, and limitations of cPOCUS has significantly improved following the theoretical module implementation. Similarly, a review of 23 studies has proved that short programs enable most learners to achieve competency (15).

Students were provided with a recorded demonstration video that explains the details of image acquisition and serves as a reference for their later use. Video-based instruction could be used as a supplement to hands-on training or set as the main teaching method for online mentoring. Combining simulation and virtual curriculum has been documented to improve both acquisition and interpretation of cPOCUS for undergraduate students (16). The use of recorded images facilitated visual training on normal as well as common pathological conditions such as pericardial effusion and myocardial dysfunction. These conditions represent life-threatening emergencies that often need prompt diagnosis and immediate management. The students demonstrated significant proficiency gains in 3 of the 5 assessed practical skills, namely, obtaining all views, showing all intended structures, and correct view interpretation. However, their performance was more modest regarding image clarity and the comprehensive visualization of all required anatomical structures. This is well-expected as image acquisition involves a steeper learning curve and requires extensive practice. These skills could be further improved through the implementation of longitudinal programs emphasizing spaced reinforcement and direct clinical application. While students had demonstrated significant improvement in short-term skills, their intermediate and long-term skills retention warrant further evaluation. Sustaining POCUS competency is essential to ensure that graduating students possess the skills needed during their internship. A POCUS training study involving preclinical medical students showed a moderate attrition in students’ skills just 8 weeks following the initial training. This decline underscores the need for a continuing longitudinal program to mitigate skill decay and ensure long-term retention (17). Despite the relatively short training period (1 hour online and 4 hours in person), most students reported feeling confident in performing and interpreting cPOCUS. The optimum duration required to achieve proficiency in POCUS skills has been investigated in many studies. A systematic review of 23 studies revealed that the learners achieved near-perfect agreement with expert echocardiographers after a period of 6 h, and substantial agreement was achievable with as little as 3 h of training (15). Such findings indicate that US training can be delivered effectively within a compact time frame, further supporting the feasibility of its inclusion in medical curriculum.

The current study initially aimed to include the application of cPOCUS by students in the hospital settings, targeting the highest level- level 4- of the Miller’s Pyramid framework. However, this target could not be achieved due to hospital policies and regulations. These findings align with a scoping review that included over 600 articles, which highlighted the scarcity of high-level assessments in undergraduate POCUS training (18).

### 4.1 Limitations

This study has several limitations that should be interpreted within the context of an exploratory educational intervention. The single-group pre–post design does not allow for direct comparison with a control group, which limits attribution of observed improvements exclusively to the intervention. Participation was voluntary, which may have resulted in a cohort with higher baseline interest or motivation toward ultrasound learning. The sample was relatively small and drawn from a single institution, which may limit broader applicability across different educational settings. Outcomes were measured immediately following the module, providing insight into short-term learning gains but not longer-term retention or transfer of skills. Although a structured checklist was used for practical assessment, formal evaluation of inter-rater reliability was not undertaken. In addition, the assessment focused on simulated and workshop-based performance rather than real clinical application due to institutional constraints. Finally, measures of confidence and satisfaction were based on self-reports and should be interpreted alongside objective performance outcomes.

## 5 Conclusion

This study reveals a significant gap in undergraduate exposure to cardiac point-of-care ultrasound, despite students’ strong awareness of its clinical importance and their eagerness to learn. The introduction of a focused, short-term teaching module led to meaningful improvements in theoretical understanding and measurable advances in basic practical skills, with most students reaching high levels of performance and confidence. These results support the feasibility of integrating structured cPOCUS training into the clinical curriculum, even with limited time and resources. Future efforts should aim to incorporate ongoing training, evaluate skill retention over time, and facilitate supervised clinical applications to enhance competency development.

## Data Availability

The raw data supporting the conclusions of this article will be made available by the authors, without undue reservation.
